# Unmanned Aerial Vehicle-Based Compressed Data Acquisition for Environmental Monitoring in WSNs

**DOI:** 10.3390/s23208546

**Published:** 2023-10-18

**Authors:** Cuicui Lv, Linchuang Yang, Xinxin Zhang, Xiangming Li, Peijin Wang, Zhenbin Du

**Affiliations:** 1 School of Computer and Control Engineering, Yantai University, Yantai 264005, China; 2 Central Research Institute, Yantai Jereh Oilfield Services Group Co., Ltd., Yantai 264003, China; 3 School of Environment and Materials Engineering, Yantai University, Yantai 264005, China

**Keywords:** environmental monitoring, wireless sensor networks, unmanned aerial vehicle, data compression

## Abstract

With the increasing concerns for the environment, the amount of the data monitored by wireless sensor networks (WSNs) is becoming larger and the energy required for data transmission is greater. However, sensor nodes have limited storage capacity and battery power. The WSNs are faced with the challenge of handling larger data volumes while minimizing energy consumption for transmission. To address this issue, this paper employs data compression technology to eliminate redundant information in the environmental data, thereby reducing energy consumption of sensor nodes. Additionally, an unmanned aerial vehicle (UAV)-assisted compressed data acquisition algorithm is put forward. In this algorithm, compressive sensing (CS) is introduced to decrease the amount of data in the network and the UAV serves as a mobile aerial base station for efficient data gathering. Based on CS theory, the UAV selectively collects measurements from a subset of sensor nodes along a route planned using the optimized greedy algorithm with variation and insertion strategies. Once the UAV returns, the sink node reconstructs sensory data from these measurements using the reconstruction algorithms. Extensive experiments are conducted to verify the performance of this algorithm. Experimental results show that the proposed algorithm has lower energy consumption compared to other approaches. Furthermore, we employ different data reconstruction algorithms to recover data and discover that the data can be better reconstructed in a shorter time.

## 1. Introduction

Wireless sensor networks (WSNs) are composed of a large number of wireless sensor nodes. Each sensor node has the ability to sense the current environment, communicate with neighboring nodes, and perform local computation. Due to the characteristics of low power consumption and low cost, the WSNs have a wide range of applications in the field of environmental monitoring. But the environmental monitoring involves more areas and the data are larger. This means that sensor nodes need to collect a large amount of data and send data to the sink node through wireless communication for processing and analysis. Sensor nodes are usually battery powered, and the computation and communication capabilities are very limited. With sensor nodes scattered over a wide area, massive data transmission can easily cause network congestion and consume more energy. Especially in monitoring areas with complex environments or that are inaccessible to humans, it is difficult to replenish energy, and the data collection in WSNs becomes challenging.

Unmanned aerial vehicles (UAVs) have high flexibility, and they can quickly reach sensor nodes to collect data without being restricted by terrain and geography. With the development of information technologies, UAVs show greater application promise in environmental monitoring. In contrast to traditional data collection methods, UAVs can cover a larger range, overcome obstacles and collect dispersed sensory data. However, the energy of UAVs is also limited, so data compression is used to reduce the energy required for data transmission. In traditional data compression methods, encoding and decoding are more complex, which can increase the computational burden on sensor nodes and shorten the lifetime of the network. Therefore, how to effectively realize compressed data acquisition is an important topic in UAV-assisted WSNs.

## 2. Related Work

The compressive sensing (CS) theory is a data dimensionality reduction method that converts data from high-dimensional space to low-dimensional space. This method can reconstruct original data with the minimal information loss, thus ensuring data accuracy [1]. In recent years, the CS-based data gathering problem has been studied widely. Karaku et al. studied the energy consumption model and theoretically analyzed the energy efficiency of the CS theory in data acquisition of WSNs [2]. Fazel et al. exploited random sensing and random access for long-term monitoring of the underwater WSNs geographic environment [3]. Chen et al. investigated compressive data collection in multi-attribute scenarios [4]. Similarly, He et al. studied the multi-attribute data gathering in heterogeneous WSNs [5], which exploited Hankel matrices and spatio-temporal correlation of sensory data for data collection. Wang et al. addressed the data aggregation problem in a dynamic WSN. When a new node joined the network, they only optimized the corresponding column vector of the new node rather than regenerated the measurement matrix [6]. Xiang et al. combined the compressed data aggregation and the Minimum Spanning Tree (MST) to minimize the energy consumption of the network [7]. The CS encoding was performed only when the packets that the sensor nodes sent were not smaller than the number of measurements. Compared with traditional data acquisition, the throughput was significantly improved under low power consumption. In [8], the interest node for CS was selected based on the sparse random matrix and their random projections were transmitted to the sink node through the MST structure. Based on cluster technology, Sun et al. put forward a sparse sampling scheme [9], which randomly selected nodes to perform the sensing task, and sent data to the gateway node through the cluster heads. Later, the UAVs were combined with the cluster technology to transmit data in WSNs [10].

In [11], Unmanned Aerial Systems (UAS) dynamically cooperated with the WSNs for effective data collection. The experimental results validated that energy consumption was reduced compared to that of traditional multi-hop data gathering methods. In [12], the wake-up schedule of sensor nodes and the trajectory of UAV were jointly optimized. Likewise, the user scheduling and the route design of the UAV were combined to minimize the data loss in large-scale WSNs [13]. Particle swarm optimization (PSO) algorithm was used for 3-D path planning of the UAV in [14]. Liu et al. took the time-constrained data collection of the UAV in environmental monitoring systems into account and formulated the UAV trajectory planning problem as an optimization problem of the UAV flight speed, hovering position and access sequence [15]. A successive convex approximation (SCA) method and genetic algorithm (GA)-based algorithm was developed to solve this problem. Qadir et al. analyzed the different metaheuristic algorithms and proposed a dynamic group-based co-optimization algorithm for UAV path optimization in a disaster situation [16].

Based on the above analysis, we summarize the related work in Table 1. Though compressive data gathering in WSNs has achieved a large number of significant results, data gathering in the UAV-assisted WSNs needs to meet the requirement of high energy efficiency and high compression efficiency, and the energy consumption of the UAV and sensor nodes must be considered simultaneously. Our study was inspired by the reference [10], which designs a highly sparse measurement matrix for data compression, enabling the UAV to visit only a subset of nodes. This article represents an extended version of our previous work [17,18]. The difference is that this article employs two pre-designed strategies for the UAV’s path planning, and investigates the performance of data monitoring using different sensing matrices and reconstruction algorithms. The main contributions are as follows.

- The CS theory is combined with UAV technology to realize efficient data collection in WSNs. To be specific, the CS theory separates encoding and decoding into measurement and reconstruction. Based on the CS theory, the sensor node only uses the simple multiplication and addition operations to implement data compression, while data reconstruction is carried out on the powerful sink node, improving the compression efficiency. In addition, the UAV is used as a mobile aerial base station to transmit data and further improves the efficiency of data collection;
- The minimization problem of energy consumption in a UAV-based data collection system is formulated as an optimization problem of the hovering locations of the UAV and path planning. For this problem, we propose a UAV-assisted compressed data acquisition algorithm. This algorithm makes use of the CS theory to determine the data collection locations of the UAV and optimizes greedy algorithm to obtain the optimal path;
- In terms of compressed data acquisition, the results of the experiment demonstrate that the proposed algorithm reduces the energy consumption of the system compared with the benchmark algorithms, such as genetic algorithm, PSO algorithm, and simulated annealing algorithm. Furthermore, the sensing matrix in the proposed algorithm can improve the data monitoring accuracy.

**Table 1:** Overview of the data collection-related work in WSNs.

Surveys	Objective	Method
Karaku et al. [2]	analyze the energy efficiency of the CS theory on the network lifetime.	build a unified computation and communication energy model, and construct the MIP framework.
Fazel et al. [3]	save energy and bandwidth.	employ the CS theory and random channel access.
Chen et al. [4]	decrease the measurements.	exploit correlations among attributes.
He et al. [5]	improve the recovery accuracy.	utilize the low-rank block Hankel matrix to exploit the inherent correlation among multi-attribute data.
Wang et al. [6]	reduce the energy consumption of the new nodes added in dynamic WSNs.	make use of the CS-based data aggregation.
Xiang et al. [7]	realize the high-fidelity data collection.	develop diffusion wavelet as the sparsifying basis and compressed data aggregation.
Ebrahimi et al. [8]	prolong the network lifetime.	exploit compressive data gathering based on the MSTP.
Sun et al. [9]	reduce the energy consumption and improve the robustness.	use the cluster-based topology for data transmission and the sparsest random sampling
Ebrahimi et al. [10]	implement energy-efficient data collection in dense WSNs.	use the UAVs and the clustered tree for compressive data gathering
Martinez-de Dios et al. [11]	improve the performance of data collection and prolong the lifetime of the nodes.	utilize the dynamic cooperation of the UAS and WSN for data collection.
Zhan et al. [12]	minimize the maximum energy consumption of all SNs.	jointly optimize the SNs’ wake-up schedule and the UAV’s trajectory.
Wang et al. [13]	minimize the data loss.	join the user scheduling and the UAV’s trajectory planning.
Yu et al. [14]	perform tasks efficiently with the UAVs.	use hybrid PSO algorithm to plan the UAVs’ path.
Liu et al. [15]	minimize the mission completion time of the UAV.	optimize the UAV’s flying speeds, hovering positions and visiting sequence through the SCA and GA-based algorithms
Qadir et al. [16]	plan the collision-free path of the UAV for pre-disaster assessment.	analyze different meta-heuristic algorithms and propose Dynamic Group-Based Cooperative Algorithm.

The rest of this article is organized as follows. In Section 3, we introduce the system model and describe a UAV-assisted compressed data acquisition algorithm in detail. Section 4 demonstrates the performance of the proposed algorithm through the experiments. Section 5 concludes this paper.

## 3. System Model and Method

This section first introduces the CS theory, and then describes the system model of the UAV and WSN. Finally, a UAV-assisted compressed data acquisition algorithm is presented.

### 3.1. CS Theory

In CS theory, when *x* has a sparse characteristic under a basis, it can be represented as Equation (1).

(1) x=ΨΘ

where Ψ is the sparsifying basis with *N* rows and *N* columns, and Θ is a coefficient vector. If the number of non-zero elements in Θ is *k*, then *x* is regarded as *k*-sparse and the sparsity is *k*. If the elements in Θ that are sorted in decreasing order of magnitude decrease quickly, *x* is approximately sparse, and the numerical sparsity is used as an alternative to the sparsity [19].

In a WSN with *N* sensor nodes, if each sensor node is marked as $s_{i}$(1≤i≤N) and the corresponding sensory data are $x_{i}$, then the *N* nodes’ sensory data can be written as a vector *x* = ${\left[x_{1},x_{2},...,x_{i},...,x_{N}\right]}^{T}$. When *x* is sparse or approximately sparse in the basis Ψ, the CS theory can be utilized to collect the measurements *y* = ${\left[y_{1},y_{2},...,y_{i},...,y_{M}\right]}^{T}$ by Equation (2).

(2) y=Φx=ΦΨΘ

where Φ is the measurement matrix with *M* rows and *N* columns, *M* is the number of measurements. Since *M* is much smaller than *N*, Equation (2) is an under-determined equation, and *x* has infinite solutions. Given the sensing matrix ΦΨ and the measurements *y*, the CS theory states that *x* can be recovered by solving the $l_{0}$-norm minimization problem in Equation (3).

(3) $$\hat{\Theta }=argmin{∥\Theta ∥}_{0}\;s.t.\;y=\Phi \Psi \Theta$$

As the problem in Equation (3) is NP-hard, two categories of solution algorithms have been extensively studied. One category is greedy algorithm, such as OMP, StOMP, CoSaMP and SP. The other one is convex optimization algorithm, which transforms the $l_{0}$-norm minimization problem into the $l_{1}$-norm minimization problem in Equation (4), and solves by linear programming, such as BP. According to these algorithms, we can obtain $\hat{\Theta }$ and then reconstruct the *N* nodes’ sensory data $\hat{x}$ by Equation (5).

(4) $$\hat{\Theta }=argmin{∥\Theta ∥}_{1}\;s.t.\;y=\Phi \Psi \Theta$$

(5) $$\hat{x}=\Psi \hat{\Theta }$$

### 3.2. The UAV-Based System Model

**Figure 1:**
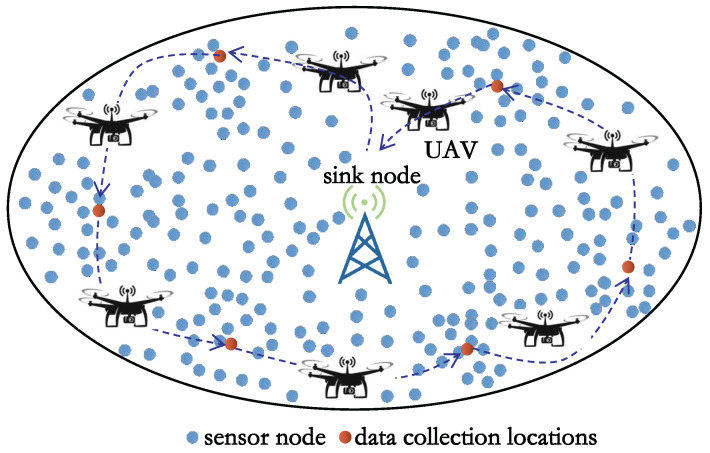
The UAV-based system model.

The system we consider contains a WSN and a UAV, as shown in Figure 1. The WSN consists of a large number of sensor nodes and one sink node $s_{0}$. The UAV stops above at the sink node. After receiving the data acquisition command, the UAV leaves the sink node to collect data.

When the UAV arrives at the specified locations, it communicates with sensor nodes through the air-to-ground communication mode [20]. The average data rate *R* between them is

(6) $$R=B\log_{2}\left(1+\frac{P}{{\bar{P}}_{Loss}N_{0}}\right),$$

where *B* is the bandwidth, *P* is the sensor nodes’ transmitting power, $N_{0}$ is the noise power of the spectral density, and ${\bar{P}}_{Loss}$ is the average path loss defined as Equation (7).

(7) $${\bar{P}}_{Loss}=p_{LoS}\left(\tau +\eta_{LoS}\right)+\left(1-p_{LoS}\right)\left(\tau +\eta_{NLoS}\right),$$

where $\eta_{LoS}$ and $\eta_{NLoS}$ are the mean value of the excessive path loss in Line-of-Sight (LoS) and Non-LoS (NLoS) links, respectively. τ is the free-space path loss with the exponent ξ, the carrier frequency *f*, the speed of light *c* and the communication distance *d*.

(8) $$\tau =10\xi \mathrm{lg}(\frac{4\pi fd}{c}),$$

$p_{LoS}$ is the LoS probability, and its value depends on the environment and the elevation angle θ of the UAV with regard to sensor nodes.

(9) $$p_{LoS}=\frac{1}{1+\alpha \exp(-\beta [\theta -\alpha ])},$$

(10) $$\theta =\frac{180}{\pi }\sin^{-1}\left(\frac{H}{d}\right),$$

In Equation (9), α and β are the parameters that are related to the urban environment. *H* in Equation (10) is the height of the UAV above the data collection locations.

When the UAV hovers at the designated location $L_{i}$, the sensor nodes start to send data. If $s_{j}$ transmits data to the UAV and the data volume is $D_{s_{j}}$, then its energy consumption $E_{s_{j}}$ is expressed as Equation (11) [20,21,22]. In this paper, each element $\phi_{ij}$ of the measurement matrix Φ only includes zero and one. When the UAV collects the data of $s_{j}$, the element $\phi_{ij}$ is set as one, otherwise, $\phi_{ij}$ is zero. Therefore, the energy consumption $E_{s}$ of the WSN is written as Equation (12).

(11) $$E_{s_{j}}=\frac{PD_{s_{j}}}{R},$$

(12) $$E_{s}=\sum_{i=1}^{M}\sum_{j=1}^{N}E_{s_{j}}\phi_{ij},$$

The UAV consumes energy in both the hovering and flying phases. When the UAV hovers at the location $L_{i}$, the energy consumption for hovering is

(13) $$E_{L_{i}}=\sum_{i=1}^{N}\frac{D_{L_{i}}}{R}\left(P_{hov}+P_{com}\right),$$

(14) $$P_{hov}=\sqrt{\frac{{\left(mg\right)}^{3}}{2\pi r^{2}n\rho }},$$

where $D_{L_{i}}$ is the data volume received by the UAV, $P_{hov}$ is the hovering power, $P_{com}$ is the communication power, *m* is the UAV’s mass, *g* is the acceleration of gravity on earth, *n* is the number of the propellers, the radius is *r*, and ρ is the air density.

When the UAV flies with the speed *v* from $L_{i}$ to $L_{j}$, its energy consumption is

(15) $$E_{L_{ij}}=\frac{d_{L_{ij}}}{v}\left(P_{hov}+P_{mov}\right),$$

(16) $$P_{mov}=\frac{v}{v_{max}}\left(P_{max}-P_{idle}\right)+P_{idle},$$

where $d_{L_{ij}}$ is the Euclidean distance between $L_{i}$ and $L_{j}$, $P_{mov}$ is the horizontal movement power. $v_{max}$ is the maximum speed, $P_{max}$ and $P_{idle}$ are the hardware power of the UAV flying at full speed and in idle state, respectively.

Based on the above analysis, we obtain the UAV’s energy consumption $E_{UAV}$ in Equation (17).

(17) $$E_{UAV}=E_{s_{0}L_{i}}+\sum_{i=1}^{M}E_{L_{i}}+\sum_{i=1,j\neq i}^{M}I_{ij}E_{L_{ij}}+E_{L_{j}s_{0}},$$

The terms $E_{s_{0}L_{i}}$ and $E_{L_{j}s_{0}}$ indicate the energy consumption of the UAV leaving the sink node to the first location $L_{i}$ and returning to the sink node from the last location $L_{j}$, respectively. *I* is a matrix with *M* rows and *N* columns. Its element is labeled as $I_{ij}$, which signifies whether the UAV moves from $L_{i}$ to $L_{j}$. If there is a path of the UAV between $L_{i}$ and $L_{j}$, the value of $I_{ij}$ is one, otherwise, $I_{ij}$ is zero.

The objective of this paper is to minimize the system’s energy consumption *E* in Equation (18), where ω is the weight of $E_{UAV}$ and $E_{s}$.

(18) $$E=\omega E_{UAV}+\left(1-\omega \right)E_{s},$$

### 3.3. Compressed Data Acquisition Algorithm

The system model indicates that the UAV hovers at each data collection site to collect data. Once the data collection sites are determined, the next step is to plan the path of the UAV to reach these locations. The detailed steps are described in Algorithm 1.
**Algorithm 1:** the UAV-assisted compressed data acquisition algorithm**Input:**  Historical data of *N* sensor nodes, the nodes’ locations and the reconstruction accuracy.**Output:**  the measurements *y*.1: Obtain the sparsifying basis based on the eigenvalue decomposition of historical data.2: Estimate the sparsity *k* of sensory data.3: Determine the number *M* of measurements based on the reconstruction accuracy.4: Get *M* data acquisition locations and the corresponding measurement matrix Φ.5: Utilize greedy algorithm to obtain a sequence of the *M* locations.6: Conduct two types of operations (variation and insertion), run *T* times for each operation and obtain a sequence of the *M* locations.7: The sink node dispatches the UAV to collect data from the *M* locations.8: After finishing data collection, the UAV carries the measurements *y* back to the sink node.

The CS theory requires sensory data to have a sparse representation in a basis. Therefore, the first step of the algorithm is to construct the sparsifying basis by means of eigenvalue decomposition and its complexity is *O*($N^{3}$). The second step is to estimate the sparsity, and it has a lower bound of the complexity *O*($N^{2}$log*N*). The third step determines the value of *M* based on the CS theory in order to guarantee the reconstruction accuracy of the data. After completing these steps, the sink node needs to determine the *M* data collection locations of the UAV. According to Equation (12), if a greater number of sensor nodes send data to the UAV at each data collection site, it results in a larger value of $E_{s}$. Consequently, in the fourth step, each sensor node is selected as a data collection site with a probability of M/N. The UAV hovers above these sites for data gathering and generates a highly sparse measurement matrix Φ, which has a computational complexity of *O*(*N*).

From Equations (15) and (17), it can be observed that the energy consumption of the UAV increases with an increase in its flight path length. When the *M* data collection sites are fixed, the minimization problem stated in Equation (17) transforms into a Traveling Salesman Problem. Due to the ease of implementation, greedy algorithm is employed to solve this problem and the time complexity is *O*(*M*log*M*). To further minimize the total path length of the UAV, this paper optimizes the greedy algorithm using Variation and Insertion (VI) operations in steps five and seven, where the VI operations have a complexity of *O*(MT). After collecting the measurements *y*, the UAV returns to the sink node for data recovery in the last step.

## 4. Results

In this section, three experiments are conducted to demonstrate the UAV-assisted compressed data acquisition algorithm. In the first experiment, six hundred sensor nodes are placed in the 1000 m × 1000 m area and the sink node is at the center of the area. The experimental parameters of the UAV-assisted system model are shown in Table 2. The data volume $D_{s_{j}}$ of each sensor node $s_{j}$ is 300 bits. This experiment mainly observes the performance of greedy algorithm with different strategies in terms of energy consumption and gives the comparison results of the four algorithms (i.e., GA, PSO, SA and Greedy algorithm with VI). The second experiment provides the data recovery results of the proposed algorithm when the reconstruction algorithms are OMP, CoSaMP, SP and BP, respectively. In the third experiment, the eight sensing matrices in previous compressive data gathering studies are compared with that in the proposed algorithm when the BP algorithm is used for data reconstruction.

In the data collection experiment, GA, PSO, SA and Greedy algorithm with VI are used for the path planning of the UAV. These algorithms are described below.

- The GA algorithm searches for the optimal solutions by simulating the natural evolutionary process. The basic steps include coding, generating an initial population, calculating a fitness function, selection, crossover, mutation, generating the next generation of population and decoding. In this experiment, the coding corresponds to the order in which the UAV visits the data collection points. The population size and the maximum number of iterations is set to 2000, the partial-mapped crossover and mutation rates are 0.8 and 0.1. In the selection process, the fitness function is the path length, it takes the inverse and then performs a roulette selection. The mutation is to randomly select two cities and swap their positions. The decoding is the path of the UAV.
- The core idea of the SA algorithm is to accept, with some probability, a solution that is worse than the current one, and then continue the search with this worse solution. During the search process, different weights are assigned to the three neighborhood structures and a roulette wheel is employed to choose a neighborhood structure. The maximum number of iterations in the inner loops is 15 and it is 300 in the outer loops.
- The PSO algorithm belongs to a kind of evolutionary algorithm. The basic steps include the initialization of particle positions and velocities, the calculation of the fitness function values, individual extremes and population extremes, and their update. Like SA, it starts from a random solution and finds the optimal solution through the iterations. In the experiment, the number of the particles is 500, and the number of iterations is 2000. The quality of the solution is evaluated through the fitness function. Compared with the rules of the GA algorithm, the PSO algorithm does not include crossover and mutation operations.
- Greedy algorithm with VI is the path planning method of the UAV in the proposed algorithm. The value of *T* in the experiment is set as 2 ×$10^{5}$. The initial path of the UAV is first planned by greedy algorithm, then the variation operation and the insertion operation are performed for optimization.

In the data reconstruction experiments, the OMP algorithm is a greedy algorithm. It selects the columns of the measurement matrix in a greedy iterative manner such that the chosen columns are maximally correlated with the current redundancy vector. The CoSaMP algorithm and the SP algorithm are the improvement of the OMP algorithm. The SP algorithm only adds *k* new candidates in each iteration, while the CoSaMP algorithm adds 2k vectors. This makes the SP algorithm more efficient than the CoSaMP algorithm. In the convex optimization algorithms, the most commonly used method is BP, which utilizes the linear programming methods to solve the $l_{1}$-norm optimization problem in Equation (4).

### 4.1. Comparison of Data Gathering Algorithms

**Figure 2:**
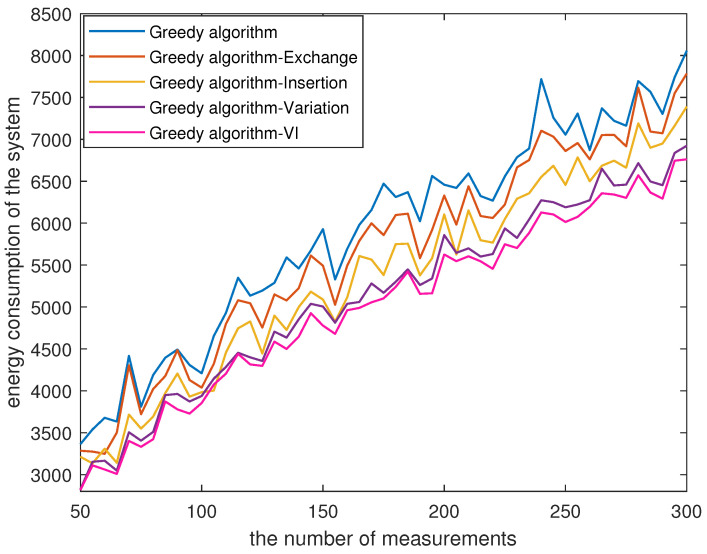
The energy consumption of greedy algorithm with different strategies.

When the number *N* of sensor nodes is 600, Figure 2 illustrates the energy consumption curves corresponding to the utilization of different strategies in a greedy algorithm for planning the UAV’s path. From Figure 2, it can be seen that the curves exhibit an increasing trend with the increment of *M*, indicating that larger values of *M* can result in more sensor nodes transmitting data to the UAV, and consequently increases the energy consumption. Furthermore, we observe that conducting insertion after variation can further reduce the energy consumption of the system.

Generally, as the value of *M* is larger, the UAV is required to visit more sensor nodes and cover a longer path, resulting in higher energy consumption for the system. When the value of *M* changes from 50 to 100, Table 3 lists the UAV’s path length and the running time of four algorithms. From the numerical results, it can be seen that the proposed algorithm achieves a shorter path length compared with the other three algorithms. In terms of the running time, the proposed algorithm is slower than the SA algorithm, but faster than the GA algorithm and PSO algorithm.

**Figure 3:**
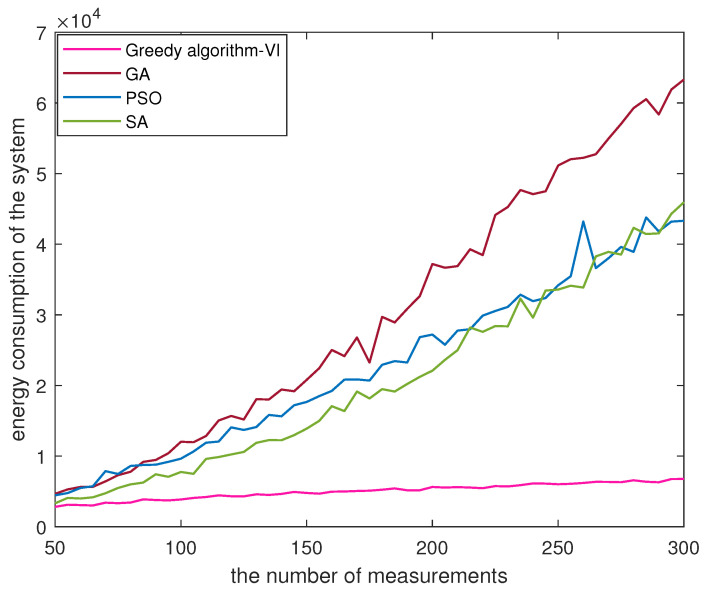
The energy consumption of the four algorithms.

According to Equations (15) and (17), the energy consumption of the system can be influenced by the path length of the UAV. To better observe the trend in energy consumption, we vary the value of *M* from 50 to 300 and present the comparative results of the four algorithms in Figure 3, with ω set at 0.5. In Figure 3, it is evident that energy consumption of the GA algorithm is the largest among the four algorithms, reaching 6.33 ×$10^{4}$ J. The PSO algorithm and the SA algorithm show the energy consumption ranging from 3.3 ×$10^{3}$ J and 4.6 ×$10^{4}$ J. When the value of *M* is smaller, the SA algorithm consumes less energy than the PSO algorithm. Compared with the other three algorithms, greedy algorithm with VI demonstrates a lower energy consumption below 6.8 ×$10^{3}$ J, indicating its ability to save energy for the same value of *M*.

### 4.2. Analysis of Data Monitoring Performance

Following the Algorithm 1, the measurements *y* are acquired by the sink node and then utilized for data recovery using the CS reconstruction algorithms. In the CS theory, the relative error ε serves as a metric for evaluating data recovery performance. In this paper, the value of ε also represents data monitoring performance and it is defined as follows.

(19) $$\varepsilon =\frac{{∥x-\hat{x}∥}_{2}}{{∥x∥}_{2}}$$

where *x* represents the sensory data of *N* sensor nodes and $\hat{x}$ represents the recovered data.

**Figure 4:**
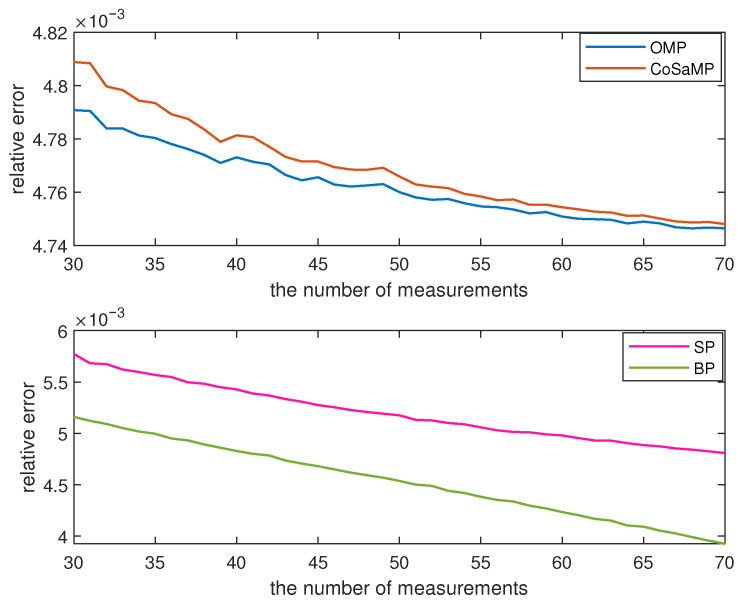
The data monitoring performance of the proposed algorithm.

**Figure 5:**
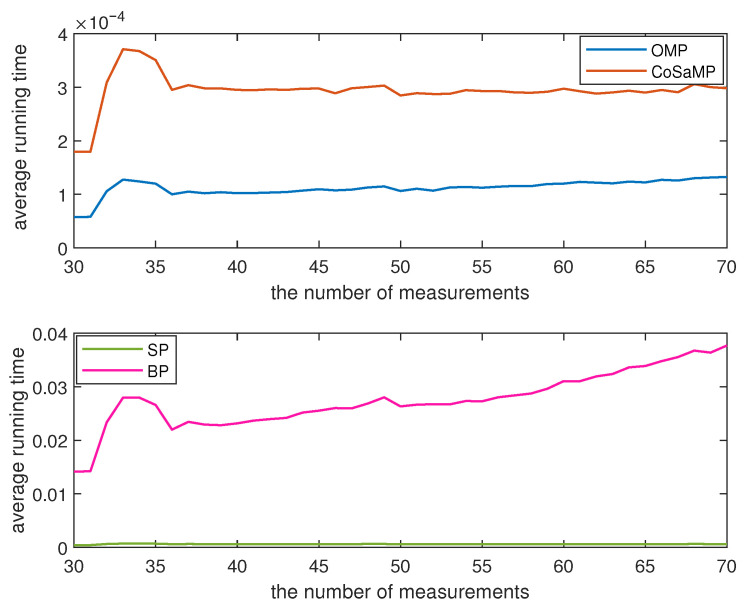
The running time of data reconstruction.

In this experiment, we select 128 sensor nodes from Green Orbs [25] and utilize their sensory data for testing purposes. For each *M* value, ranging from 30 to 70, two thousand experiments are conducted and the average value of ε is illustrated in Figure 4. It can be observed from Figure 4 that the value of ε gradually decreases with an increase in *M*. Among the four reconstruction algorithms, the OMP algorithm and the CoSaMP algorithm exhibit similar data recovery performance. However, the relative error of the BP algorithm is at least 0.5596 ×$10^{-3}$ smaller than that of the SP algorithm. Additionally, when the value of *M* is less than 40, both the OMP algorithm and the CoSaMP algorithm outperform the SP algorithm and the BP algorithm in terms of relative error reduction. With the increase of *M*, the BP algorithm surpasses the other three algorithms, decreasing from 0.0048 to 0.0039. The average running time of the data reconstruction is depicted in Figure 5. The maximum running time of the OMP algorithm and CoSaMP algorithm is 1.3255 ×$10^{-4}$ s and 3.7124 ×$10^{-4}$ s, respectively. Meanwhile, the SP algorithm and BP algorithms are separately 4.2622 ×$10^{-4}$ s and 0.0141 s. In summary, if a larger value of *M* is chosen, the BP algorithm exhibits advantages in terms of data reconstruction, but it takes more time than the other three algorithms.

### 4.3. Comparison of Data Monitoring Performance

**Figure 6:**
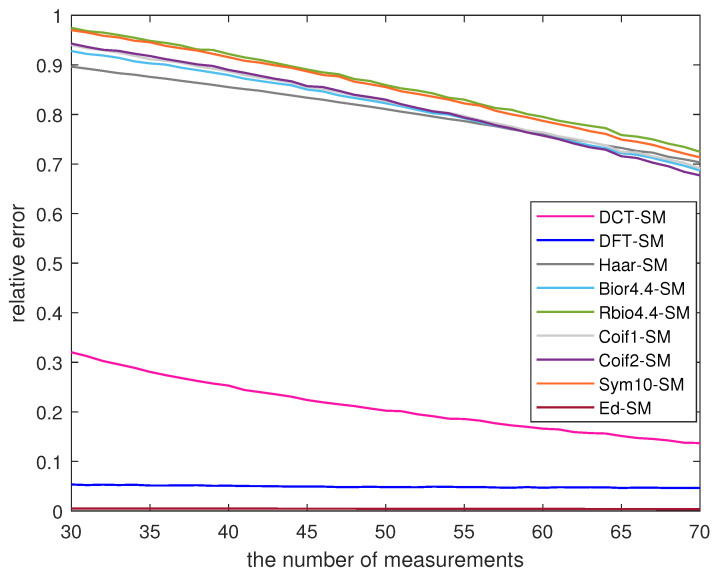
The reconstruction results of different sensing matrices.

When the reconstruction algorithms are the same, the performance of data recovery can be affected by the sensing matrix. In this experiment, we consider nine sensing matrices, where the measurement matrix is identical to that used in the proposed algorithm, denoted as SM. The sparsifying bases are obtained through the eigenvalue decomposition (Ed), transformations such as DCT and DFT, as well as a series of wavelet bases. When the BP algorithm is used for data recovery, Figure 6 displays the reconstruction results of the nine sensing matrices.

When the value of *M* increases, Figure 6 demonstrates a decreasing trend in the value of ε. In Ed-SM, the maximum and minimum values of ε are 0.0051 and 0.0039. For DCT-SM and DFT-SM, the maximum values of ε are 0.3201 and 0.0534, respectively. However, the remaining six sensing matrices yield the values of ε not less than 0.6771. In contrast, DCT-SM, DFT-SM and Ed-SM exhibit superior data monitoring performance compared to the other six sensing matrices. Furthermore, the relative error of DFT-SM is smaller than DCT-SM. But when compared with DFT-SM, Ed-SM in the proposed algorithm can reduce the relative error by at least 0.0423.

### 4.4. Discussion

In the UAV-assisted WSNs, the proposed algorithm requires the UAV to just collect data from a small amount of sensor nodes and the path of the UAV is quickly planned by greedy algorithm with VI. Based on the experimental results above, we observe that the energy consumption of the GA algorithm is the largest among the four algorithms, and the energy consumption of greedy algorithm with VI is less than the other three algorithms. The energy consumption of the PSO algorithm and the SA algorithm is between the GA algorithm and greedy algorithm with VI. This means that the proposed algorithm can reduce the energy consumption of the system when the UAV and the CS theory are combined to gather data in the environment. After the UAV finishes data gathering and returns, the sink node receives the compressed data from the UAV and starts to recover data. We carry out the experiment to verify the performance of data monitoring and discover that the OMP algorithm and the CoSaMP algorithm can obtain better data monitoring results than the BP algorithm and the SP algorithm when the value of *M* is smaller. But if the value of *M* is larger, the BP algorithm and the SP algorithm are superior to the OMP algorithm and the CoSaMP algorithm in terms of data monitoring. In addition, we compare the data monitoring performance of nine sensing matrices. Through the numerical results, we see that the Ed-SM in the proposed algorithm can make the relative error less than 0.0051.

## 5. Conclusions

This paper mainly studies the data acquisition issues of environmental monitoring in WSNs and how the UAV is utilized to reduce energy consumption of data gathering. Considering that the data of sensor nodes within a neighborhood have strong spatial correlation, the paper adopts the CS theory to compress the data in the network, constructs the system model and proposes a UAV-assisted compressed data acquisition algorithm. Compared with three benchmark algorithms, the experimental results show that the proposed algorithm can shorten the total trip of the UAV during data gathering and decrease energy consumption of the system. In terms of data monitoring, the Ed-SM in the proposed algorithm can reduce the relative error of data recovery compared to other methods, and obtain better data monitoring results.

## Figures and Tables

**Table 2:** The experimental parameters of the UAV-assisted system model.

Parameter	Value	Parameter	Value	Parameter	Value
*m*	500 g [22]	*n*	4 [22]	*r*	20 cm [22]
*v*, $V_{max}$	15 m/s [23]	$P_{max}$, $P_{idle}$	5 w, 0 w [22]	*H*	50 m [20]
$P_{com}$	0.0126 w [23]	*P*	21 dBm/Hz [23]	$N_{0}$	−174 dBm/Hz [23]
*B*	1 MHz [20]	$\eta_{LoS}$, $\eta_{NLoS}$	1 dB, 20 dB [24]	*f*	2 GHz [23]
ξ	3 [23]	α	10 [23]	β	0.03 [23]

**Table 3:** The comparison results of the algorithms when the value of *M* ranges from 50 to 100.

The Value of *M*	Algorithms	The UAV’s Path Length (×$10^{4}$)	Running Time (s)
50	Greedy algorithm-VI	0.5789	2.2914
	GA	0.9602	22.5102
	PSO	0.9155	49.5505
	SA	0.6829	0.0804
60	Greedy algorithm-VI	0.6393	2.1403
	GA	1.0887	20.7948
	PSO	0.9819	55.3686
	SA	0.8401	0.0397
70	Greedy algorithm-VI	0.6288	2.2115
	GA	1.1590	22.5486
	PSO	1.1289	60.8934
	SA	0.8214	0.0320
80	Greedy algorithm-VI	0.6175	2.4702
	GA	1.1628	24.9415
	PSO	1.1803	24.9415
	SA	0.8569	0.0325
90	Greedy algorithm-VI	0.6985	2.7317
	GA	1.3224	26.2906
	PSO	1.6216	73.7638
	SA	0.9751	0.0363
100	Greedy algorithm-VI	0.6837	2.7370
	GA	1.5032	27.2675
	PSO	1.5390	78.3294
	SA	1.1294	0.0363

## Data Availability

The data in this paper can be obtained from the corresponding author.
